# PERSONALITY AND PREDEATH GRIEF IN FAMILY CAREGIVERS OF PERSONS WITH DEMENTIA

**DOI:** 10.1093/geroni/igae098.0328

**Published:** 2024-12-31

**Authors:** Elisabeth McLean, Lauren Elliott, Sydnie Schneider, Volker Neugebauer, Jonathan Singer

**Affiliations:** Texas Tech University, Lubbock, Texas, United States; Texas Tech University, Lubbock, Texas, United States; Texas Tech University, Lubbock, Texas, United States; Texas Tech University Health Sciences Center, Lubbock, Texas, United States; Texas Tech University, Lubbock, Texas, United States

## Abstract

Caring for a family member with Alzheimer’s Disease or Alzheimer’s Disease Related Dementias (AD/ADRD) is associated with psychosocial difficulties, including pre-death grief (PDG; grief symptoms that can develop while an individual with a chronic, life-limiting illness is still living). PDG can be debilitating and is a robust predictor of prolonged grief disorder (PGD) once the person with AD/ADRD passes away. Past research has demonstrated several personality factors related to PGD in bereaved individuals; for example, low extraversion and agreeableness and high neuroticism have been found to confer greater risk of developing prolonged grief disorder after a death. However, it is presently unknown whether personality factors are related to the development of PDG prior to the death. Data were analyzed from a sample of family caregivers of individuals with AD/ADRD (Mage = 68.68, SD age = 11.89). Participants answered a battery of questions regarding pre-death grief (Prolonged Grief—12; PG-12), perceived social support (Oslo Social Support Scale; OSSS), and the NEO Five Factor Inventory (NEO-FFI-3). Bivariate correlations revealed non-significant relationships between all personality dimensions except for neuroticism (r =.441, p <.001). Additionally, a multiple linear regression examining the impact of neuroticism and perceived social support on PDG revealed a significant main effect of neuroticism (F(2, 52) = 6.53, p =.002, R2 =.17) on pre-death grief. Thus, family caregivers who have high levels of neuroticism may be at increased risk of experiencing pre-death grief and clinical intervention might be more warranted for this population.

